# Meta‐analysis and field synopsis of genetic variants associated with the risk and severity of acute pancreatitis

**DOI:** 10.1002/bjs5.50231

**Published:** 2019-12-03

**Authors:** F. F. van den Berg, M. A. Kempeneers, H. C. van Santvoort, A. H. Zwinderman, Y. Issa, M. A. Boermeester

**Affiliations:** ^1^ Department of Surgery Amsterdam University Medical Centre, University of Amsterdam Amsterdam the Netherlands; ^2^ Department of Clinical Epidemiology, Biostatistics and Bioinformatics Amsterdam University Medical Centre, University of Amsterdam Amsterdam the Netherlands; ^3^ Department of Surgery St Antonius Hospital Nieuwegein the Netherlands; ^4^ Department of Surgery University Medical Centre Utrecht Utrecht the Netherlands

## Abstract

**Background:**

Genetic risk factors can provide insight into susceptibility for acute pancreatitis (AP) and disease progression towards (infected) necrotizing pancreatitis and persistent organ failure. The aim of the study was to undertake a systematic review of the genetic evidence for AP.

**Methods:**

Online databases (MEDLINE, Embase, BIOSIS, Web of Science, Cochrane Library) were searched to 8 February 2018. Studies that reported on genetic associations with AP susceptibility, severity and/or complications were eligible for inclusion. Meta‐analyses were performed of variants that were reported by at least two data sources. Venice criteria and Bayesian false‐discovery probability were applied to assess credibility.

**Results:**

Ninety‐six studies reporting on 181 variants in 79 genes were identified. In agreement with previous meta‐analyses, credible associations were established for *SPINK1* (odds ratio (OR) 2·87, 95 per cent c.i. 1·89 to 4·34), *IL1B* (OR 1·23, 1·06 to 1·42) and *IL6* (OR 1·64, 1·15 to 2·32) and disease risk. In addition, two novel credible single‐nucleotide polymorphisms were identified in Asian populations: *ALDH2* (OR 0·48, 0·36 to 0·64) and *IL18* (OR 1·47, 1·18 to 1·82). Associations of variants in *TNF*, *GSTP1* and *CXCL8* genes with disease severity were identified, but were of low credibility.

**Conclusion:**

Genetic risk factors in genes related to trypsin activation and innate immunity appear to be associated with susceptibility to and severity of AP.

## Graphical abstract

A comprehensive systematic review and meta‐analysis of the genetic evidence for acute pancreatitis was conducted, and identified credible associations of *SPINK1*, *ALDH2*, *IL1B*, *IL6* and *IL18* with the risk of acute pancreatitis. A lack of replication studies precludes definite conclusions to be drawn regarding the role of genetics in severity and severe clinical phenotypes of acute pancreatitis.

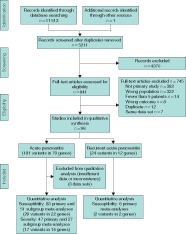

Genes affecting trypsin activation and innate immunity important

## Abstract (es)

**Antecedentes:**

Los factores de riesgo genético pueden contribuir a determinar la susceptibilidad para desarrollar una pancreatitis aguda (*acute pancreatitis*, AP) y de su progresión a pancreatitis necrotizante (infectada) e insuficiencia orgánica crónica. Nuestro objetivo fue revisar de forma sistemática la evidencia genética de la pancreatitis aguda.

**Métodos:**

Se revisaron las bases de datos electrónicas (MEDLINE, Embase, BIOSIS, Web of Science, Cochrane Library) hasta febrero de 2018. Se incluyeron estudios que presentaban información de las asociaciones genéticas y la susceptibilidad de AP, gravedad y/o complicaciones. Se realizó un metaanálisis de las variantes genéticas descritas en al menos dos fuentes. Se aplicaron los criterios de Venecia y la probabilidad bayesiana de falsa alarma para la valoración de la credibilidad.

**Resultados:**

Se identificaron 96 estudios que analizaron 181 variantes en 79 genes. De acuerdo con un metaanálisis previo, se establecieron asociaciones creibles con el riesgo de enfermedad para SPINK1 (razón de oportunidades, *odds ratio*, OR 2,87, i.c. del 95% 1,89‐4,34), IL1B (OR 1,23, i.c. del 95% 1,06‐1,42) e IL6 (OR 1,64, i.c. del 95% 1,15‐2,32). Además, en poblaciones asiáticas, se identificaron dos nuevos polimorfismos de nucleótico único (SNP) creibles en ALDH2 (OR 0,48, i.c. del 95% 0,36‐0,64) e IL18 (OR 1,47, i.c. del 95% 1,18‐1,82). En cuanto a la gravedad de la enfermedad se identificaron variantes en los genes TNF, GSTP1 y CXCL8, pero de baja credibilidad en función de nuestra evaluación.

**Conclusión:**

Los factores de riesgo genéticos en genes relacionados con la activación de la tripsina y la inmunidad innata parecen ser estar asociados con la susceptibilidad y gravedad de la pancreatitis aguda.

## Introduction

Acute pancreatitis (AP) is a common inflammatory disease of the pancreas with an incidence of 13–45 per 100 000 population in Western countries^1^. Most patients experience a mild disease course with hospital discharge within 1 week, but around 20 per cent develop severe AP. Pancreatic necrosis occurs in about 30 per cent of patients, and severe complications such as secondary bacterial infection of (peripancreatic) necrosis and (persistent) organ failure lead to mortality rates of up to 30 per cent^2^.

Biliary disease and alcohol are the leading causes of AP, and account for 70 per cent of cases; other causes are hypertriglyceridaemia, hypercalcaemia, iatrogenic (mostly related to endoscopic retrograde cholangiopancreatography and drugs) and autoimmune disease, and some are classified as idiopathic^3^. The risk of developing AP in patients with asymptomatic gallstone disease or heavy alcohol consumers is 2–3 per cent, indicating a complex multifactorial pathogenesis^4^, ^5^. As well as host factors such as smoking and diabetes, genetic variants are suspected to play an important role in disease susceptibility, outcome and progression^6^. Improved understanding of genetic risk factors may help to identify patients at risk of AP, severe complications and progression to chronic pancreatitis.

Since the discovery of the causal variant of hereditary pancreatitis in the *PRSS1* gene by Whitcomb and colleagues^7^, two decades of mostly candidate gene genetic association studies have been initiated. Other disease‐causing variants (such as *SPINK1*, *CTRC*, *CASR* and *CFTR* 8) were discovered, and stimulated the initiation of genetic association studies in patients with AP. However, the majority of identified associations could not be replicated in similar or other populations, probably because most studies lacked statistical power owing to insufficient sample sizes. Summarization and analysis of current knowledge is needed^9^.

Field synopsis and meta‐analysis are powerful tools to integrate genetic data from a large field and identify credible genetic associations^10^, ^11^, ^12^. In the present review, all genetic data associated with AP were systematically collected and summarized. In addition, credibility of the evidence was assessed by applying the Venice criteria^9^ and the Bayesian false‐discovery probability (BFDP) method^13^. The aim was to provide a framework for clinicians and researchers to guide genetic research, and the development of clinical diagnostic tools and personalized therapeutic interventions.

## Methods

This systematic study was conducted by following a protocol, and is concordant with the Updated Guidance on Human Genome Epidemiology Reviews and Meta‐Analysis of Genetic Associations^14^, and the PRISMA guidelines^15^ for systematic reviews and meta‐analysis (*Appendix* *S1*, supporting information).

### Data sources and searches

A systematic search of five online databases (MEDLINE, Embase, BIOSIS, Web of Science, Cochrane Library) was performed, using the keywords and index terms ‘pancreatitis’ combined with ‘mutation*’, ‘polymorphism*’ and ‘variant*’, and applying the limit ‘human’. After removal of duplicates, identified articles were screened for eligibility in two steps: first by title and/or abstract independently by two authors, and second by reading of the full text, and selected based on predefined inclusion and exclusion criteria. The references of identified studies and reviews were cross‐checked for additional studies. In case of disagreement, consensus was resolved by discussion.

### Study selection

Genetic association studies (case–control and cohort) associated with AP and recurrent AP in humans published in a peer‐reviewed journal before 8 February 2018 that met the criteria were included with no restriction on language. Genome‐wide association studies were considered, but not identified. Studies reporting on other than bi‐allelic markers were excluded from quantitative analyses. Studies reporting on tropical, hereditary or autoimmune pancreatitis are considered as chronic pancreatitis, and were excluded. Abstracts, case reports, economic evaluations, *in vitro* and animal studies were excluded. Studies reporting exclusively on paediatric patients (age below 18 years) were also excluded, because of differences in aetiology^16^.

Published meta‐analyses of identified variants were considered for comparison. Studies with identical, or largely overlapping, sets of data were compared, and the largest data set was included in the meta‐analysis; when data were identical, the first published study was included.

### Data extraction and quality assessment

Data extraction was performed separately by two authors, and differences were resolved by discussion. As well as study identifiers such as first author, year of publishing, journal and language, the following demographic data were extracted: study location and design, inclusion and exclusion criteria, sample size, reported outcomes, studied aetiology, ethnic background of patients and control subjects, source of controls, age and sex.

The following data points for each variant were extracted: Single Nucleotide Polymorphism database (dbSNP) identifier number, Human Gene Nomenclature Committee symbol and name, type of genetic variation, position and genotype distribution. Genotype counts were calculated when genotype frequencies and numbers of cases and controls were reported. If only allele frequencies were available, genotype counts were calculated assuming distribution according to the Hardy–Weinberg equilibrium (HWE).

The corresponding author was contacted when genotype counts were not presented in the article or could not be calculated, or if an inconsistency was noticed. They were asked to supply raw data (genotype distribution) or clarification. The data points were excluded for quantitative analysis because of insufficient data if no, or only a partial, response was received after two attempts.

The credibility of significant associations was assessed according the Venice criteria, based on the published recommendations of the HuGENet Working Group^9^. The Venice criteria involve a three‐point grading system, assessing the amount of evidence (power), replication and protection from bias. To correct for multiple testing, the BFDP method was applied. The overall assessment of credibility was, consistent with the recommendations, based on both the BFDP and Venice grade, and was rated strong (A), moderate (B) or weak (C).

### Statistical analysis

All analyses were performed using R studio version 3.3.2^17^. The packages meta and metafor^18^ were used for meta‐analysis and funnel plotting. Because mode of inheritance is mostly unknown in complex traits, the primary meta‐analysis was conducted using the allelic contrast model, in which allele frequencies of the major and minor allele are compared (A *versus* a). In addition, meta‐analysis were performed using dominant (AA + Aa *versus* aa) and recessive (AA *versus* Aa + aa) genetic models. Unless stated otherwise, the effect of the minor allele (based on the Ensemble database) was investigated^19^. For extracted variants that had been reported on by multiple studies, summary odds ratios (ORs) and 95 per cent c.i. were calculated using a random‐effects model (DerSimonian and Laird). Between‐study heterogeneity was calculated with the Q test and *I* ^2^ statistic. Deviation from HWE was calculated systematically, and defined as *P* < 0·050 using a χ^2^ test with 1 degree of freedom. Funnel plot analysis and the Harbord statistical test^20^ was performed for significant associations to detect possible publication bias and small‐study effects. Sensitivity analysis was performed for significant associations excluding studies that deviated from HWE. Where possible, subgroup meta‐analysis for severity and ethnicity was done.

## Results

**Figure 1:**
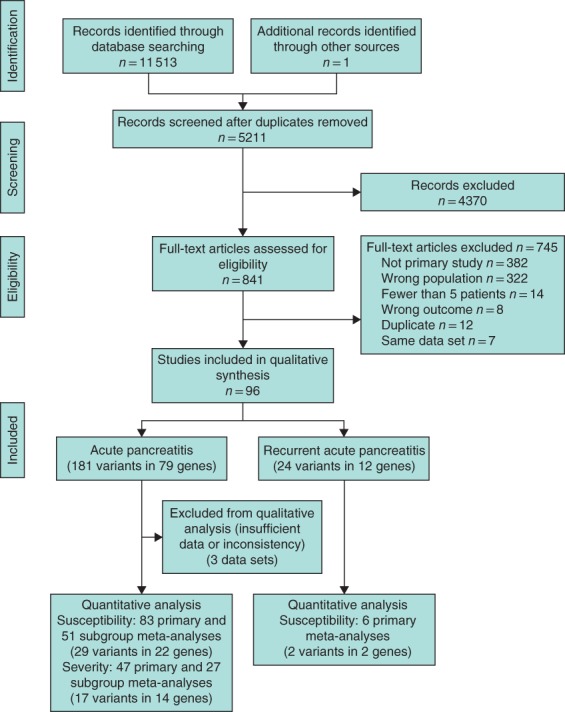
Flow diagram of study selection and quantitative analysis

The search resulted in 841 studies potentially eligible for inclusion, of which 96 articles met the inclusion and exclusion criteria (*Fig*. 1; *Table* *S1*, supporting information). Eighty‐seven studies reported on AP, six studies on recurrent disease and three on both. This resulted in a total of 181 studied variants in 79 genes for AP and 24 variants in 12 genes for recurrent AP. Eight published meta‐analyses of genetic variants associated with AP were identified, reporting on nine single‐nucleotide polymorphisms (SNPs) in nine different genes (*Table* 1). The remaining SNPs in the present review had not previously been subjected to meta‐analysis.

**Table 1:** Genes and variants associated with susceptibility for acute pancreatitis compared with previously published meta‐analyses

		Present meta‐analysis					Previous meta‐analyses				
Gene	Variant	No. of cases	No. of controls	No. of studies	Outcome	Credibility*	Reference	No. of cases	No. of controls	No. of studies	Outcome†
*SPINK1*	rs17107315	1493	2595	9	+	A	Joergensen *et al*.^21^	1135	2822	8	+
*IL1B*	rs1143634	1301	1171	6	+	C	Yin *et al*.^22^	519	388	3	+
*CXCL8 (IL8)*	rs4073	1770	1990	9	+	C	Yin *et al*.^22^	503	758	5	+
*ACE*	rs4646994	770	4878	4	−		Fang *et al*.^23^	245	1455	3	+
*CCL2*	rs1024611	470	562	4	−		Fang *et al*.^24^	567	562	4	+
*CD14*	rs2569190	1328	1195	6	−		Yuan and Wang^25^	1211	932	5	−
*IL10*	rs1800896	1738	1691	8	−		Yin *et al*.^22^	339	243	2	−
*TLR4*	rs4986790	994	794	4	−		Zhou *et al*.^26^	1255	998	6	−
*TNF*	rs1800629	1335	1076	6	−		Yin *et al*.^27^	1006	782	6	−

* Assessed by Venice criteria, based on sample size, heterogeneity among studies, and risk of bias.

† Reported by original author.

+, positive association; −, no association.

To provide a comprehensive overview, the variants extracted from the studies are presented per gene category (*Table* *S2*, supporting information). Together, the studies included 18 138 patients and 32 227 control subjects. Two‐thirds of the studies were performed in a Caucasian population; the remaining populations were Asian (30·5 per cent), Hispanic/Latin American (1·7 per cent) or of mixed origin (0·7 per cent). Further study characteristics are presented in *Appendix* *S1* (supporting information).

Subgroup analysis based on severity of the disease course in AP was done in 45 studies. Other clinical outcome measures such as pancreatic necrosis, infectious and systemic complications, mortality and need for surgery were reported for four, 12, 41, four and three variants respectively (*Table* *S3*, supporting information).

Thirty‐one of the 205 extracted variants were reported by at least two articles and were subsequently selected for meta‐analysis. Three data sets were excluded due to insufficient or inconsistent data. A mean of 750 (median 512 (i.q.r. 325)) patients and 940 (median 652 (i.q.r. 418)) controls per variant were included in the primary meta‐analyses. Results of the primary and subgroup meta‐analyses, sensitivity analyses and credibility assessments are presented in *Tables* *S3* *–* *S8* (supporting information). Nine nominal significant associations in six variants (genes *SPINK1, ALDH2, IL1B, IL6, GSTT1* and *IL18*) were identified using the allelic contrast genetic model (*Table* 2). Additional meta‐analyses using dominant and recessive genetic models revealed six more significant associations (*Table* 3) in five additional variants (genes *IL18, IL1B, CCL2, CXCL8* and *IL10)*. After credibility assessment, five and three associations were rated as having strong and moderate evidence respectively. The significant associations are discussed in more detail below.

**Table 2:** Nominal significant associations for variants identified for meta‐analysis using the allelic contrast genetic model with credibility factors in (recurrent) acute pancreatitis

									Effect size		Heterogeneity		Credibility		
Gene	Variant	Group	Minor allele	Model	Ethnicity	No. of cases	No. of controls	No. of studies	OR	*P*	*I* ^2^ (%)	*P*	BFDP	Venice	Overall
*SPINK1*	rs17107315	AP	C	Allelic	Mixed	1493	2595	9	2·87 (1·89, 4·34)	6·3 × 10^−7^	0·0	0·493	0·055	AAA	A
*SPINK1*	rs17107315	AP	C	Allelic	White	1085	1687	5	2·49 (1·55, 3·98)	1·5 × 10^−4^	0·0	0·244	0·529	AAA	A
*SPINK1*	rs17107315	RAP	C	Allelic	Mixed	233	1300	3	7·51 (3·20, 17·64)	3·7 × 10^−6^	15	0·389	0·75	AAA	A
*SPINK1*	rs17107315	RAP	C	Allelic	White	136	758	2	5·65 (2·69, 11·88)	4·9 × 10^−6^	0·0	0·847	0·664	BAA	B
*ALDH2*	rs671	AP	A	Allelic	Asian	350	272	2	0·48 (0·36, 0·64)	3·3 × 10^−7^	0·0	0·415	0·008	BAA	B
*IL1B*	rs1143634	AP	A	Allelic	Asian	997	899	4	1·23 (1·03, 1·45)	0·018	0·0	0·318	0·788	AAC	C
*IL6*	rs1800795	AP	C	Allelic	Asian	607	607	2	1·22 (1·00, 1·47)	0·045	0·0	0·726	0·882	AAA	C
*GSTT1*	Null	AP	Null	Allelic	White	575	804	4	0·66 (0·44, 0·99)	0·045	41·8	0·138	0·907	AAA	C
*IL18*	rs1946518	AP	T	Allelic	Asian	325	418	2	1·25 (1·00, 1·57)	0·049	0·0	0·781	0·905	BAA	C

Values in parentheses are 95 per cent confidence intervals. OR, odds ratio; BFDP, Bayesian false‐discovery probability; AP, acute pancreatitis; RAP, recurrent acute pancreatitis.

**Table 3:** Nominal significant associations for variants identified for meta‐analysis using dominant or recessive genetic models with credibility factors in (recurrent) acute pancreatitis

									Effect size		Heterogeneity		Credibility		
Gene	Variant	Group	Minor allele	Model	Ethnicity	No. of cases	No. of controls	No. of studies	OR	*P*	*I* ^2^ (%)	*P*	BFDP	Venice	Overall
*IL1B*	rs16944	AP	A	Dominant	Asian	857	861	4	1·23 (1·06, 1·42)	0·005	0·0	0·519	0·622	AAA	A
*IL6*	rs1800795	AP	C	Recessive	Asian	607	607	2	1·64 (1·15, 2·32)	0·006	0·0	0·685	0·754	AAA	A
*IL18*	rs187238	AP	G	Dominant	Asian	325	418	2	1·47 (1·18, 1·82)	4·9 × 10^−4^	0·0	0·328	0·234	BAA	B
*CCL2*	rs1024611	RAP	G	Dominant	Mixed	302	652	2	2·19 (1·23, 3·89)	0·008	58·6	0·120	0·876	BCA	C
*CXCL8 (IL8)*	rs4073	AP	T	Recessive	Mixed	1770	1990	9	1·29 (1·06, 1·57)	0·010	57·7	0·015	0·760	ACC	C
*CXCL8 (IL8)*	rs4073	AP	T	Recessive	White	368	400	4	1·19 (1·01, 1·40)	0·034	0·0	0·300	0·885	BAC	C
*IL10*	rs1800872	AP	C	Dominant	Mixed	830	802	4	1·17 (1·01, 1·35)	0·040	0·0	0·992	0·880	AAA	C
*IL1B*	rs1143634	AP	T	Recessive	Mixed	1301	1171	6	1·41 (1·00, 1·99)	0·048	26·9	0·295	0·906	ABC	C

Values in parentheses are 95 per cent confidence intervals. OR, odds ratio; BFDP, Bayesian false‐discovery probability; AP, acute pancreatitis; RAP, recurrent acute pancreatitis.

### Variants associated with disease risk

#### *Serine protease inhibitor Kazal type 1 (SPINK1)*

Premature intrapancreatic trypsinogen activation leads to autodigestion and is believed to be a key initiating process in the pathogenesis of pancreatitis^3^, ^28^. Transcriptions of the *SPINK1* gene in the pancreas function as a trypsin inhibitor, and loss‐of‐function variants are believed to increase autoactivation of trypsinogen^29^. Twenty variants in the *SPINK1* gene were described in ten different studies. Only two of the ten studies reported an association of AP susceptibility with the N34S variant (c.101A>G, rs17107315). Functionality of the variant remains unclear^30^. The present meta‐analysis of nine studies (1493 patients, 2595 controls) identified an association in the allelic model with susceptibility for AP (OR 2·87, 95 per cent c.i. 1·89 to 4·34; *P* = 6·3 × 10^−7^) (*Table* 2). This association remained robust following subgroup analysis of five studies of Caucasians (OR 2·49, 1·55 to 3·98; *P* = 1·5 × 10^−4^), but not of Asians. The association was rated as strong, indicating a highly credible finding.

A Caucasian population of 72 patients with recurrent disease and 670 controls showed a significant association with the N34S variant^31^, and has been confirmed in a Japanese study^32^ but not in an Italian population^33^. Meta‐analysis of these studies confirmed this association in the overall population (3 studies) (OR 7·51, 95 per cent c.i. 3·20 to 17·64; *P* = 3·7 × 10^−6^) and the Caucasian population (2 studies) (OR 5·65, 2·69 to 11·88; *P* = 4·9 × 10^−6^) (*Table* 2). Two articles^34^, ^35^ reported on the functional c.194+2T>C (IVS3+T>C, rs148954387) variant, but found no association. The present meta‐analysis of 210 patients and 707 control subjects confirmed this result.

Meta‐analysis of variants of other members of the trypsin family of serine proteases and peptidases, such as *PRSS1* and *PRSS2*, showed no correlation with disease occurrence or phenotype (*Tables* *S3–S14*, supporting information).

#### *Cytokines: interleukins* 1β, *6 and 18, and C‐C motif chemokine ligand 2*

Interleukins are produced mainly by macrophages and lymphocytes, and act as proinflammatory or anti‐inflammatory communication molecules between cells of the immune system. Cytokines are likely causal candidates and have therefore been investigated extensively. Positive associations with AP susceptibility were reported in genes *IL1B*, *IL1RA*, *CXCL8* (*IL8*), *IL10* and *IL18* (*Table* *S2*, supporting information).

*IL1B* codes for interleukin (IL) 1β, one of the proinflammatory cytokines that is increased during an episode of AP^36^. The functional 511 C/T (g.4490 T>C, rs16944) promoter variant enhances gene expression of *IL1B* 37, and has been associated with the severity of the episode^38^, ^39^. Meta‐analysis of four studies (all Asian populations) including 857 patients and 861 controls showed a correlation with AP risk in the dominant genetic model (OR 1·23, 95 per cent c.i. 1·06 to 1·42), but not in allelic contrasts. Between‐study heterogeneity was low, and the association was rated as strong with a BFDP of 0·622 (*Table* 3). There was no association of the synonymous variant rs1143634 in allelic contrasts in six studies of patients with mixed ethnicity (4 Asian, 2 Caucasian). However, when performing ethnic subgroup analysis, a correlation was found for the Asian populations (OR 1·23, 1·03 to 1·45; *P* = 0·018) (*Table* 2). Meta‐analysis of the recessive genetic model found positive associations in both Asian and mixed populations. Significance was lost in all associations when excluding one study in which the control group deviated from HWE.

IL‐6 is another proinflammatory cytokine that is correlated with disease severity^36^. The −174G>C intronic variant (g.4880C>G, rs1800795) demonstrated lower expression of *IL6 in vitro*, and is associated with susceptibility to juvenile rheumatoid arthritis^40^. Pooled analyses of two studies (both Asian populations) in 607 patients and controls were associated with susceptibility to AP in both the allelic contrast (OR 1·22, 95 per cent c.i. 1·00 to 1·47; *P* = 0·045) (*Table* 2) and the recessive genetic model (OR 1·64, 1·15 to 2·32; *P* = 0·006) (*Table* 3). Credibility of the associations was weak and strong respectively, based on BFDP values of 0·882 and 0·754.

Two functional intronic variants of *IL18* have been studied in AP. The −607C/A (g.4383A>C, rs1946518) and − 137G>C (g.4853G>C, rs187238) variants have been shown to increase IL‐18 mRNA levels in expression analyses assays^41^. Meta‐analyses of the rs1946518 variant in two studies reached nominal significance (OR 1·25, 95 per cent c.i. 1·00 to 1·57; *P* = 0·049), but was deemed weak evidence after BFDP correction (*Table* 2). The rs187238 variant was associated with AP risk in the dominant genetic model only (OR 1·47, 1·18 to 1·82; *P* = 4·9 × 10^−4^), and was rated as moderate evidence because of the limited sample size (*Table* 3).

C‐C motif chemokine ligand 2 (CCL2), also referred to as monocyte chemoattractant protein 1 (MCP‐1), is involved in the migration of inflammatory cells, and is expressed during pancreatic inflammation^42^. Papachristou *et al*.^43^ found an association of the functional rs1024611 variant with severe AP. This was not replicated in patients with AP in another Caucasian population; however, an over‐representation of the G allele was found in patients with recurrent disease^33^. Meta‐analysis did not confirm an association with AP. However, this was established in two studies of patients with recurrent disease in the dominant genetic model (OR 2·19, 95 per cent c.i. 1·23 to 3·89; *P* = 0·008). Owing to heterogeneity, the credibility was rated as weak.

Meta‐analyses of other interleukin variants resulted in weak associations, owing to failure to pass correction for multiple testing (*IL1B* rs1143634), between‐study heterogeneity (*IL10* rs1800872), or both (*CXCL8* rs4073).

#### *Mitochondrial aldehyde dehydrogenase (ALDH2)*

Four variants in genes related to the ethanol metabolism pathway have been investigated in Asian populations. Variants in these genes are common in Asian populations and associated with susceptibility to alcoholism, alcohol sensitivity and oesophageal cancer.

The *2 allele of *ALDH2* (g.42421G>A, rs671) was found significantly less often in patients with alcoholic pancreatitis, but not in those with a biliary aetiology^44^, ^45^. When pooling patients with both aetiologies from two studies, a protective association was found for AP risk with the minor *2 allele (OR 0·48, 95 per cent c.i. 0·36 to 0·64; *P* = 3·3 × 10^−7^) in allelic contrasts (*Table* 2). The credibility score was moderate (BFDP 0·008, Venice grade BAA) owing to the relatively small sample size. Meta‐analysis of variants in other genes related to ethanol metabolism, such as *ADH2*, *ADH3* and *CYP2E1*, did not show significant associations with disease risk or severity.

#### *Antioxidant enzymes: glutathione S‐transferase*

The antioxidant enzyme glutathione S‐transferase (GTS) protects tissues from free radical injury and has four classes: alpha (GTSA), mu (GSTM), pi (GSTP) and theta (GSTT)^46^. The effect of ten variants of these genes on AP susceptibility was investigated. The effect of four functional alleles in three studies was studied, but only one SNP in the *GSTM1* gene showed an association with AP susceptibility^47^. Meta‐analysis could not confirm this, but found instead an overrepresentation of the null allele of *GSTT1* in controls (OR 0·66, 95 per cent c.i. 0·44 to 0·99; *P* = 0·045); however, this was rated as weak evidence owing to the likelihood of being a false‐positive association based on a BFDP value of 0·907 (*Table* 2).

### Variants associated with severity of acute pancreatitis

Reported associations with disease severity and complications were assessed systematically; 17 variants reported on by more than two articles were identified (*Tables* *S9* *–* *S14*, supporting information). Meta‐analysis showed three significant associations in the genes *CXCL8*, *GSTP1* and *TNF* (tumour necrosis factor (TNF) α) (*Table* 4). However, the BFDP value for the *CXCL8* and *GSTP1* variants (both greater than 0·8) indicated a high likelihood of a false‐positive outcome. The BFDP value for the *TNF* variant was very low, indicating a highly significant association. However, significance was lost when sensitivity analysis excluded one study that deviated from HWE, indicating the presence of genotyping error or population stratification^47^. The credibility of all three associations was subsequently rated as weak. Nine positive associations with disease severity in the genes *TLR3*, *TLR4*, *TLR6*, *CD14*, *NFKBIA*, *PKA2G7*, *PPARG* and *SERPINE1* were found that were not replicated in another study (*Table* 5). Although the limited data on other disease phenotypes did not allow for pooled analyses, positive associations were identified for infectious complications (*TLR4*, *CD14*, *DEFB1*, *IL10*, *REN*), systemic complications (*TNF*, *TNFAIP3*, *PLA2G7*), pancreatic necrosis (*HMOX1*), mortality (*REN*) and surgery (*TLR2*). These are viable candidate genes, and replication is needed (*Table* 5).

**Table 4:** Nominal significant associations for variants identified for meta‐analysis with credibility factors comparing patients with mild and severe acute pancreatitis

									Effect size		Heterogeneity		Credibility		
Gene	Variant	Group	Minor allele	Model	Ethnicity	No. of cases	No. of controls	No. of studies	OR	*P*	*I* ^2^ (%)	*P*	BFDP	Venice	Overall
*TNF*	rs1800629	AP	A	Recessive	Mixed	703	702	6	0·54 (0·41, 0·71)	1·3 × 10^−5^	0	0·707	0·041	AAC	C
*GSTP1*	rs1695	AP	G	Recessive	White	282	125	2	1·43 (1·05, 1·93)	0·022	0	0·950	0·845	BAA	C
*CXCL8 (IL8)*	rs4073	AP	T	Dominant	Mixed	387	200	6	0·74 (0·56, 0·98)	0·039	29	0·159	0·950	BBC	C

Values in parentheses are 95 per cent confidence intervals. OR, odds ratio; BFDP, Bayesian false‐discovery probability; AP, acute pancreatitis; RAP, recurrent acute pancreatitis.

**Table 5:** Genes and variants associated with disease severity or complications of acute pancreatitis that were not replicated in another study*

Gene	Variant	Reference	Outcome	Incidence (%)	No. of cases	No. of controls	Risk allele
**Severity**							
*CD14*	rs5744455	Masamune *et al*.^34^	SAP	31	107	238	C
*TLR3*	rs3775291	Matas‐Cobos *et al*.^48^	SAP	13	36	233	C
*TLR4*	G11367C	Zhang *et al*.^49^	SAP	33	150	300	C
*TLR6*	rs7543795	Matas‐Cobos *et al*.^48^	SAP	13	36	233	A
*NFKBIA*	rs696	Zhang *et al*.^49^	SAP	33	150	300	T
*PLA2G7*	rs16874954	Ma *et al*.^50^	SAP	51	486	46	A
*PLA2G7*	rs1805017	Ma *et al*.^50^	SAP	51	48	46	A
*PPARG*	rs1801282	Zhang *et al*.^51^	SAP	33	150	300	G
*SERPINE1*	rs1799889	Tukiainen *et al*.^52^	SAP	33	150	300	5G
**Pancreatic necrosis**							
*HMOX1*	S/L	Gulla *et al*.^53^	Pancreatic necrosis	50	63	64	L
**Infectious complications**							
*CD14*	rs5744455	Masamune *et al*.^34^	Infected necrosis	9	32	314	C
*TLR4*	rs4986790	Gao *et al*.^54^	Infected necrosis	24	30	95	G
*DEFB1*	rs11362	Tiszlavicz *et al*.^55^	Infected necrosis	23	29	95	A
*DEFB1*	rs1799946	Tiszlavicz *et al*.^55^	Infected necrosis	23	29	95	C
*IL10*	rs1800896	Zhang *et al*.^56^	Septic shock	30	33	76	G
*REN*	rs5707	Skipworth *et al*.^57^	Infected necrosis	14	52	317	G
**Systemic complications**							
*TNF*	rs1799964	Bishehsari *et al*.^58^	MODS	11	23	188	C
*TNF*	rs1800630	Bishehsari *et al*.^58^	MODS	11	23	188	A
*TNF*	rs361525	de‐Madaria *et al*.^59^	Systemic complications	11	9	75	A
*TNFAIP3*	rs5029924	Liu *et al*.^60^	SIRS	35	47	88	T
*PLA2G7*	rs16874954	Ma *et al*.^50^	MODS	6	6	88	A
**Mortality**							
*REN*	rs5707	Skipworth *et al* 57	Mortality	7	36	477	C
**Surgery**							
*TLR2*	Microsatellite	Takagi *et al*.^61^	Surgery	15	30	172	S

* Reported by original author.

SAP, severe acute pancreatitis; MODS, multiple organ dysfunction syndrome; SIRS, systemic inflammatory response syndrome.

## Discussion

This systematic review and meta‐analysis of genetic association studies in AP found two genetic variants in *SPINK1* and *ALDH2* genes that showed moderate or strong credible associations with disease risk in the allelic contrasts model, and three credible associations using dominant and recessive models in *IL1B*, *IL6* and *IL18*. However, except for the *SPINK1* variants, credible associations were found only with pooled analysis of Asian populations. In addition, three weak associations with disease severity were found in *CXCL8*, *GSTP1* and *TNF* (TNFα). The application of credibility criteria ensures a high likelihood of identifying only true and robust associations.

The loss‐of‐function N34S variant (c.101A>G, rs17107315) in the *SPINK1* gene is presumed to be a disease modifier for recurrent and chronic, but not for acute, pancreatitis^6^, ^62^. However, a meta‐analysis by Joergensen and colleagues^21^ showed an association with AP risk, which remained robust in the present updated meta‐analyses of both AP and recurrent AP. The trypsin inhibitory function of *SPINK1*, the relatively large sample size of approximately 1500 patients, and the absence of significant heterogeneity indicates a reliable role in susceptibility to AP and progression to recurrent or chronic disease. Pathogenic variants in the trypsinogen gene *PRSS1* are causative for hereditary pancreatitis with a penetrance of 80 per cent^7^. Because of its known functional consequence, *PRSS1* would also be expected to be associated with AP or recurrent AP. Surprisingly, such an association was not found. However, as *PRSS1* variants are associated with early‐onset pancreatitis, they are possibly underrepresented in the present data set owing to the exclusion of studies performed exclusively in paediatric populations. Other possibilities are data paucity, lack of statistical power, and the fact that different variants were studied.

A protective association for the *ALDH2* rs671 variant was found after pooled analyses of two studies by the same authors containing 350 patients and 272 controls of Chinese origin. The functional consequence of this non‐synonymous variant in *ALDH2* is a defective enzyme involved in ethanol breakdown, and is common in Asian, but rare in non‐Asian, populations. The A allele is related to alcohol sensitivity, and shown to be protective against alcoholism and alcohol‐associated diseases, such as alcoholic pancreatitis^63^. Due to lack of appropriate control groups (alcoholics without alcoholic pancreatitis or other alcohol‐associated disease) in the original studies, it remains uncertain whether this variant has a true association with alcoholic pancreatitis, or rather is associated with a reduced alcohol intake.

The role of cytokines in the pathogenesis of AP has long been recognized^31^. Previous meta‐analyses of variants have identified associations with AP in *CXCL8* (*IL8*) and *CCL2*, but not in other cytokine genes (*IL1B, IL10* or *TNF*)^22^, ^24^, ^27^. Meta‐analysis showed significant associations of only low credibility with disease risk in *TNF, CXCL8* (*IL8*) and *GSTP1* genes. A previous meta‐analysis^24^ reported significant associations between AP risk and the G allele of the *CCL2* rs1024611 variant. The present meta‐analysis of the same studies contradicted these results. The present authors explain this discrepancy by an inconsistency in the data of one study^64^, which was verified by the original author during data assessment. In addition, the meta‐analyses performed by Fang *et al*.^24^ included patients with recurrent disease.

A variant in the *ACE* gene was previously reported as having a positive association with AP, but this was not confirmed in the present study; meta‐analysis of the *ACE* insertion–deletion (rs4646994) in 245 patients and 1455 controls showed a positive association with the insertion^23^. This meta‐analysis was updated with a large study, and the association was lost in both genetic models. In general, discrepancies between the present and former meta‐analyses can be explained by the fact that, in some cases, additional studies were included, credibility criteria were applied, and there was bias in the earlier studies based on the choice of genetic model (dominant or recessive). Here, the allelic contrasts model, which is independent of mode of inheritance, was used for the primary analysis.

**Figure 2:**
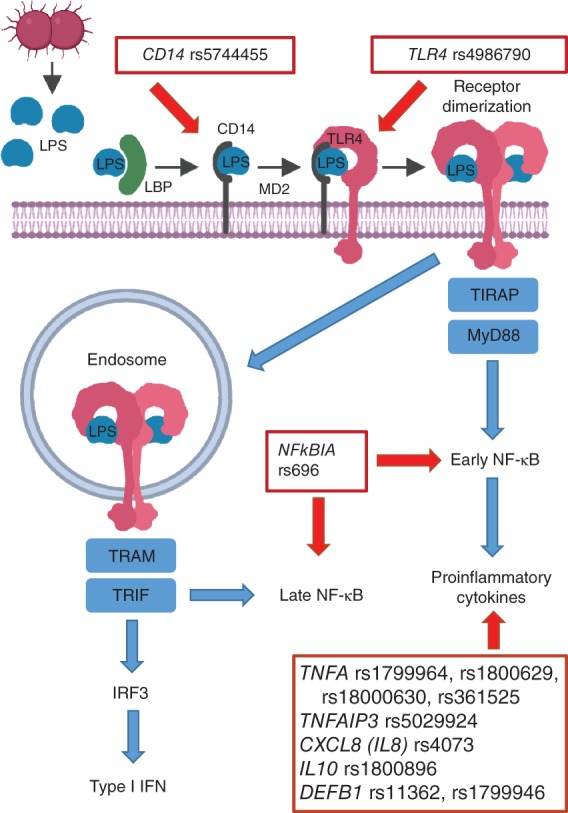
TLR4/CD14 pathway and genetic variants significantly associated with disease progression (severity or complications), identified by unreplicated primary studies or meta‐analysis CD, cluster of differentiation; LPS, lipopolysaccharide; TLR, Toll‐like receptor; LBP, lipopolysaccharide binding protein; MD2, lymphocyte antigen 96; TIRAP, Toll–interleukin 1 receptor (TIR) domain‐containing adaptor protein; MyD, myeloid differentiation primary response 88; NF, nuclear factor; TRAM, translocating chain‐associated membrane protein; TRIF, TIR‐domain‐containing adapter‐inducing interferon β; IRF, interferon regulatory transcription factor; IFN, interferon.

No credible associations with disease severity (defined by the Atlanta criteria^65^) could be established in the pooled analyses. A paucity of data relating variants to disease outcome and complications is the main explanation for the lack of credible associations. Reported unreplicated genetic variants associated with infected (peri)pancreatic necrosis^34^, ^54^, ^55^, ^56^, ^57^ and multiple organ failure syndrome^50^, ^58^, ^59^ are involved mainly in pathways of innate immunity (*Fig*. 2). Lipopolysaccharide (endotoxin), produced by Gram‐negative bacteria, is an important activator of the Toll‐like receptor 4 (TLR4) pathway that leads to innate immune system activation, the production of proinflammatory cytokines (TNFα, IL‐1β, IL‐6) and antimicrobial peptides (β‐defensins). A role has been suggested for cytokine polymorphisms in the development of severe complications^28^, ^66^; however, redundancy in these downstream inflammatory responses makes it less likely that these variants alone can account for progression to severe AP. Associated variants in these genes, together with (unidentified) variants in more upstream genes of the pathway, need replication to investigate causality in the pathogenesis of disease progression.

This review has some limitations, so the results of the present meta‐analyses should be interpreted with caution. A general limitation of synthesis of genetic association studies in complex diseases is the lack of replication and small sample sizes. This is supported by a study^67^ showing that only 3·6 per cent of associations can be replicated consistently. Usually, large sample sizes are needed to establish a causal effect. Therefore, most small‐scale studies are underpowered and at risk of not detecting true associations (type II error). This means that a non‐significant association does not necessarily mean that there is no causal relationship with the phenotype of interest. To correct for multiple testing of the variants, the authors used the BFDP method. Although this method is independent of the number of associations being evaluated, and therefore less conservative than, for example, the Bonferroni method, there is a risk of losing true associations (false‐negatives). Second, there is significant heterogeneity in the definition of severity and complications of AP. Some studies use clinical scoring systems that are frequently used by ICUs, such as Acute Physiology And Chronic Health Evaluation (APACHE) II and Ranson scores. These are, however, limited by poor sensitivity and specificity^68^, ^69^, leading to underestimation of the true effect size. Third, due to lack of data reporting in the included studies, it was often difficult to make a complete risk of bias assessment. For example, most studies did not report whether the studied population included patients with recurrent disease. Some 17 and 8 per cent of patients with a first episode of AP progress within 5 years to recurrent AP and chronic pancreatitis respectively^68^. Owing to this limitation, it is not possible to draw definitive conclusions regarding the effect of genetic variation on sentinel AP. Furthermore, only a few studies reported on methodologically important factors such as blinding of laboratory personnel, genotyping quality controls, missing data and ethnicity. In addition, details of statistical analyses were often missing; only around half of the studies mentioned whether they calculated deviation from HWE of the control group. Correction for multiple testing was used in less than half of the studies reporting on three or more variants. Finally, the source of control subjects was heterogeneous and often not specified. Control subjects are ideally sex‐ and age‐matched, and selected from the same population as the patients^70^.

Although the clinical relevance of identifying single associated genetic variants remains debatable, it can potentially provide clues about pathophysiological mechanisms and lead to the discovery of novel therapeutic targets. Furthermore, the accumulation of genetic risk factors can have a significant impact on diagnostic and prognostic strategies, as shown for other complex diseases such as Parkinson's disease^71^.

Future genetic studies need to be well designed, adequately powered, and include an extensively phenotyped patient cohort with clear definitions of aetiology. International collaboration and joint analyses will increase the power and methodological quality of genetic research. For standardization of definitions, the authors recommend using the revised Atlanta criteria^65^. Currently, there is no consensus regarding when to classify patients as having recurrent AP. Follow‐up of 5 years allows for the exclusion of patients who progress to recurrent or chronic pancreatitis, and for the identification of true causal variants for sentinel AP. High‐throughput sequencing, such as exome sequencing, has been successful for the identification of novel genetic risk factors in complex diseases, but generally requires a large sample size and a control group drawn from the same population. Variants associated with specific phenotypes (infected necrotizing pancreatitis or multiple organ dysfunction syndrome) can be identified by applying strategies that reduce the need for large sample sizes, such as selection of patients from opposing phenotypic extremes^72^, ^73^, ^74^.

Genetic risk factors in genes related to trypsin activation and innate immunity appear to be associated with AP susceptibility and severity. However, robust replication studies among genetic associations with severe clinical phenotypes are needed to push forward clinical applications.

Although open for debate, there are indications that multiple variants with a modest effect size contribute to the occurrence of disease progression processes such as pancreatic necrosis and secondary systemic or infectious complications.

## Supporting information


**Appendix S1.** Supplementary methods and results

**Table S1.** Characteristics of included studies
**Table S2.** Comprehensive overview of extracted genetic variants associated with (recurrent) acute pancreatitis, categorized by gene function
**Table S3.** Primary meta‐analysis of variants associated with susceptibility for (recurrent) acute pancreatitis
**Table S4.** Subgroup meta‐analysis of variants associated with susceptibility for (recurrent) acute pancreatitis in patients of Caucasian ethnicity
**Table S5.** Subgroup analysis of variants associated with susceptibility for (recurrent) acute pancreatitis in patients of Asian ethnicity
**Table S6.** Sensitivity analysis (excluding studies that deviate from HWE) of variants associated with susceptibility for (recurrent) acute pancreatitis
**Table S7.** Sensitivity analysis (excluding studies that deviate from HWE) of variants associated with susceptibility for (recurrent) acute pancreatitis in patients of Caucasian ethnicity
**Table S8.** Sensitivity analysis (excluding studies that deviate from HWE) of variants associated with susceptibility for (recurrent) acute pancreatitis in patients of Asian ethnicity
**Table S9.** Primary meta‐analysis of variants associated with disease severity
**Table S10.** Subgroup meta‐analysis of variants associated with disease severity in patients of Caucasian ethnicity
**Table S11.** Subgroup analysis of variants associated with disease severity in patients of Asian ethnicity
**Table S12.** Sensitivity analysis (excluding studies that deviate from HWE) of variants associated with disease severity
**Table S13.** Sensitivity analysis (excluding studies that deviate from HWE) of variants associated with disease severity in patients of Caucasian ethnicity
**Table S14.** Sensitivity analysis (excluding studies that deviate from HWE) of variants associated with disease severity in patients of Asian ethnicity
