# Supplementary material for: Meta‐analysis and field synopsis of genetic variants associated with the risk and severity of acute pancreatitis
Source: BJS Open. 2019 Dec 3;4(1):3–15. doi: 10.1002/bjs5.50231 (PMC6996643; doi:10.1002/bjs5.50231)
Supplement: Supplementary file 1 — Appendix S1. Supplementary methods and results [file BJS5-4-3-s001.docx]

**BJS5_50231**

**Meta-analysis and field synopsis of genetic variants associated with the risk and severity of acute pancreatitis**

**F. F. van den Berg^1^, M. A. Kempeneers^1^, H. C. van Santvoort^3,4^, A. H. Zwinderman^2^, Y. Issa^1^ and M. A. Boermeester^1^**

**Table S1** Characteristics of included studies

**Table S2** Comprehensive overview of extracted genetic variants associated with (recurrent) acute pancreatitis, categorized by gene function

**Table S3** Primary meta-analysis of variants associated with susceptibility for (recurrent) acute pancreatitis

**Table S4** Subgroup meta-analysis of variants associated with susceptibility for (recurrent) acute pancreatitis in patients of Caucasian ethnicity

**Table S5** Subgroup analysis of variants associated with susceptibility for (recurrent) acute pancreatitis in patients of Asian ethnicity

**Table S6** Sensitivity analysis (excluding studies that deviate from HWE) of variants associated with susceptibility for (recurrent) acute pancreatitis

**Table S7** Sensitivity analysis (excluding studies that deviate from HWE) of variants associated with susceptibility for (recurrent) acute pancreatitis in patients of Caucasian ethnicity

**Table S8** Sensitivity analysis (excluding studies that deviate from HWE) of variants associated with susceptibility for (recurrent) acute pancreatitis in patients of Asian ethnicity

**Table S9** Primary meta-analysis of variants associated with disease severity

**Table S10** Subgroup meta-analysis of variants associated with disease severity in patients of Caucasian ethnicity

**Table S11** Subgroup analysis of variants associated with disease severity in patients of Asian ethnicity

**Table S12** Sensitivity analysis (excluding studies that deviate from HWE) of variants associated with disease severity

**Table S13** Sensitivity analysis (excluding studies that deviate from HWE) of variants associated with disease severity in patients of Caucasian ethnicity

**Table S14** Sensitivity analysis (excluding studies that deviate from HWE) of variants associated with disease severity in patients of Asian ethnicity

**Appendix S1** Supplementary methods and results

***Supplementary Methods***

*Data extraction*

Variants identification numbers were identified using the Single Nucleotide Polymorphism Database (dbSNP) and Variant Name Mapper from the HuGE Navigator.^97^ The most common name was used whenever the variant could not be located in the databases. When available, reference alleles and minor allele frequencies were retrieved from the ENSEMBL database.^98^

*Credibility assessment of associations*

Study power was based on the sample size, assuming a case-control ratio of 1:1. Categorization was as follows: A) over 1000, B) 100-1000 C) less than 100. The Venice score for replication of results was based on the I2 value for heterogeneity with level A corresponding to less than 25%, level B corresponding to 25-50% and level C corresponding to values of 50% and above. Assessment of the bias component of the Venice criteria was complicated, because most articles did not supply sufficient information to make a full assessment. When publication bias was suspected, credibility of the result was graded as “C”. To correct for multiple testing we used the Bayesian false discovery probability (BFDP) at a threshold for noteworthiness set at 0.20.^99^ This means that we accept that 20% of all significant results are false, as is used elsewhere.^100-102^ Unlike other methods for multiple comparisons such as the Bonferroni, the BFDP is based on the odds ratio and 95% confidence interval and is independent from the number of comparisons. We calculated the BFDP value for the level of prior probability of finding a true gene-association of 0.05, consistent with what is used for a candidate-gene approach.

The overall assessment of credibility was, consistent with the recommendations,^103^ based on both the BFDP and the Venice grade; and was rated strong, moderate or weak. “strong” for AAA grade, “moderate” when at least one “B”, but no “C” grades were assigned, and “weak” for other grades or a BFDP above 0.8 at a prior of 0.05.

**Supplementary Results**

*Study characteristics*Five articles from the same author reported on overlapping or identical datasets^86, 104-107^. Only the largest sample size was included in the analysis^86^. Two pairs of studies were published in different languages but shared datasets, the English version was included. ^17, 22, 108, 109^

Besides English, three studies were written in Russian^13, 45, 68^, Two studies in Chinese^11, 18, 89, 90^ and one in Korean^35^. Two studies were performed in a population of patients with alcoholic pancreatitis^35, 83^, one in biliary pancreatitis^93^, one in non-biliary pancreatitis^69^ and the remaining studies in a population of mixed etiology.

Selection of the control cohort differed greatly among included studies; % (17/96) recruited from the general population or community, % (26/96) recruited patients visiting the hospital or clinic or were admitted, % (18/96) used random blood donors as controls and % (30/96) did not describe control recruitment protocol. % (6/79) did not include a control group (table 1). Around half of studies (41 / 96) matched controls for ethnicity, 2 studies were performed in a population of mixed ethnicity^2, 91^. Around half of studies reported insufficient demographic information of the control population.

Genotype quality measures such as blinding of researchers and laboratory personal for case-control status and internal genotype quality control checks were rarely reported (respectively, 4.2% and 26%). HWE of the control group was calculated in around half of studies (49/96). Only 8 studies reported that their sample size was based on a power calculation ^28, 53, 62, 67, 72, 76, 78, 80^, and 13 of 36 studies (36.1%) examining more than two variants used correction for multiple testing by Bonferroni or similar method. ^6, 48, 50, 51, 70, 82^

**References**

1. Anilir E, Ozen F, Yildirim IH, Ozemir IA, Ozlu C, Alimoglu O. IL-8 gene polymorphism in acute biliary and non biliary pancreatitis: probable cause of high level parameters? Annals of hepato-biliary-pancreatic surgery. 2017;21(1):30-8.

2. Aoun E, Muddana V, Papachristou GI, Whitcomb DC. SPINK1 N34S is strongly associated with recurrent acute pancreatitis but is not a risk factor for the first or sentinel acute pancreatitis event. Am J Gastroenterol. 2010;105(2):446-51.

3. Bagul A, Pushpakom S, Balachander S, Newman WG, Siriwardena A. The SPINK1 N34S variant is associated with acute pancreatitis. Eur J Gastroenterol Hepatol. 2009;21(4):485.

4. Balog A, Gyulai Z, Boros LG, Farkas G, Takacs T, Lonovics J, et al. Polymorphism of the TNF-alpha, HSP70-2, and CD14 genes increases susceptibility to severe acute pancreatitis. Pancreas. 2005;30(2):e46-50.

5. Bao XB, Ma Z, Gu JB, Wang XQ, Li HG, Wang WY. IL-8 -251T/A polymorphism is associated with susceptibility to acute pancreatitis. Genet Mol Res. 2015;14(1):1508-14.

6. Bhat YM, Papachristou GI, Park JS, Lamb J, Slivka A, Whitcomb DC. Functional polymorphisms of the GSTT-1 gene do not predict the severity of acute pancreatitis in the United States. Pancreatology. 2007;7(2-3):180-6.

7. Bishehsari F, Sharma A, Stello K, Toth C, O'Connell MR, Evans AC, et al. TNF-alpha gene (TNFA) variants increase risk for multi-organ dysfunction syndrome (MODS) in acute pancreatitis. Pancreatology. 2012;12(2):113-8.

8. Bishu S, Koutroumpakis E, Mounzer R, Stello K, Pollock N, Evans A, et al. The -251 A/T Polymorphism in the IL8 Promoter is a Risk Factor for Acute Pancreatitis. Pancreas. 2018;47(1):87-91.

9. Bosques-Padilla FJ, Vazquez-Elizondo G, Gonzalez-Santiago O, Del Follo-Martinez L, Gonzalez OP, Gonzalez-Gonzalez JA, et al. Hypertriglyceridemia-induced pancreatitis and risk of persistent systemic inflammatory response syndrome. Am J Med Sci. 2015;349(3):206-11.

10. Cai F, Cui N, Ma HY, Wang XL, Qiao GH, Liu DP. Interleukin-10-1082A/G polymorphism is associated with the development of acute pancreatitis in a Chinese population. International Journal of Clinical and Experimental Pathology. 2015;8(11):15170-6.

11. Cao DQ, Xiao CZ. Association between an interleukin-8 promoter polymorphism (-251A/T) and susceptibility to acute pancreatitis. [Chinese]. World Chinese Journal of Digestology. 2010;18(33):3580-3.

12. Cavestro GM, Zuppardo RA, Bertolini S, Sereni G, Frulloni L, Okolicsanyi S, et al. Connections between genetics and clinical data: Role of MCP-1, CFTR, and SPINK-1 in the setting of acute, acute recurrent, and chronic pancreatitis. Am J Gastroenterol. 2010;105(1):199-206.

13. Chantsev VA, Leonov VV. Polymorphism of gene of IL-8 (A-251T) in patients with acute pancreatitis. [Russian]. Georgian Med News. 2014(231):35-8.

14. Chao YC, Wang LS, Hsieh TY, Chu CW, Chang FY, Chu HC. Chinese alcoholic patients with esophageal cancer are genetically different from alcoholics with acute pancreatitis and liver cirrhosis. Am J Gastroenterol. 2000;95(10):2958-64.

15. Chao YC, Wang SJ, Chu HC, Chang WK, Hsieh TY. Investigation of alcohol metabolizing enzyme genes in Chinese alcoholics with avascular necrosis of hip joint, pancreatitis and cirrhosis of the liver. Alcohol and Alcoholism. 2003;38(5):431-6.

16. Chao YC, Chu HC, Chang WK, Huang HH, Hsieh TY. CD14 promoter polymorphism in Chinese alcoholic patients with cirrhosis of liver and acute pancreatitis. World J Gastroenterol. 2005;11(38):6043-8.

17. Chen WC, Nie JS. Genetic polymorphism of MCP-1-2518, IL-8-251 and susceptibility to acute pancreatitis: a pilot study in population of Suzhou, China. World J Gastroenterol. 2008;14(37):5744-8.

18. Chen XQ, Huang HG, Zhou YN, Li Y, Chen YC. The study of the relationship between interleukin gene polymorphism and the morbidity of patients with acute pancreatitis. [Chinese]. Chinese Critical Care Medicine. 2009;21(2):99-102.

19. Chi DZ, Chen J, Huang DP. Influence of interleukin-1beta and interleukin-6 gene polymorphisms on the development of acute pancreatitis. Genet Mol Res. 2015;14(1):975-80.

20. de-Madaria E, Martinez J, Sempere L, Lozano B, Sanchez-Paya J, Uceda F, et al. Cytokine genotypes in acute pancreatitis: association with etiology, severity, and cytokine levels in blood. Pancreas. 2008;37(3):295-301.

21. Farkas G, Tiszlavicz Z, Takacs T, Szabolcs A, Somogyvari F, Mandi Y. Analysis of plasma levels and polymorphisms of S100A8/9 and S100A12 in patients with acute pancreatitis. Pancreas. 2014;43(3):485-7.

22. Gao HK, Zhou ZG, Li Y, Chen YQ. Toll-like receptor 4 Asp299Gly polymorphism is associated with an increased risk of pancreatic necrotic infection in acute pancreatitis: a study in the Chinese population. Pancreas. 2007;34(3):295-8.

23. Guenther A, Aghdassi A, Muddana V, Rau B, Schulz HU, Mayerle J, et al. Toll-like receptor 4 polymorphisms in German and US patients are not associated with occurrence or severity of acute pancreatitis. Gut. 2010;59(8):1154-5.

24. Gui HB, Du XG, Fu ZH, Chen XM. Influence of interleukin-18 gene polymorphisms on acute pancreatitis susceptibility in a Chinese population. Genet Mol Res. 2016;15(3).

25. Gulla A, Evans BJ, Navenot JM, Pundzius J, Barauskas G, Gulbinas A, et al. Heme oxygenase-1 gene promoter polymorphism is associated with the development of necrotizing acute pancreatitis. Pancreas. 2014;43(8):1271-6.

26. Hofner P, Balog A, Gyulai Z, Farkas G, Rakonczay Z, Takacs T, et al. Polymorphism in the IL-8 gene, but not in the TLR4 gene, increases the severity of acute pancreatitis. Pancreatology. 2006;6(6):542-8.

27. Hucl T, Kylanpaa-Back ML, Witt H, Kunzli B, Lempinen M, Schneider A, et al. HFE genotypes in patients with chronic pancreatitis and pancreatic adenocarcinoma. Genet Med. 2007;9(7):479-83.

28. Hucl T, Kylanpaa ML, Kunzli B, Witt H, Lempinen M, Schneider A, et al. Angiotensin-converting enzyme insertion/deletion polymorphism in patients with acute and chronic pancreatitis. Eur J Gastroenterol Hepatol. 2009;21(9):1032-5.

29. Ivashchuk SI, Sydorchuk LP. Association of the genes il-4 (c-590t), TNF-alpha (g-308a), prss1 (r122h) and CFTR (delf508c) with cytolysis syndrome activity in patients with acute edematous pancreatitis. Archives of the Balkan Medical Union. 2016;51(1):41-5.

30. Jia HL, Sun PL, Lu CQ. Investigation of the association between Interleukin-10 polymorphisms and risk of acute pancreatitis in a Chinese population. Genet Mol Res. 2015;14(4):15876-81.

31. Jiang BZ, Tang L, Xue H, Liu DP. Role of IL-10 gene polymorphisms in the development of acute pancreatitis. Genet Mol Res. 2016;15(2).

32. Jiang S, Ni M, Zhang Y, Wu Y, Lu XZ. Association of IL-10 polymorphisms with acute pancreatitis. International Journal of Clinical and Experimental Medicine. 2016;9(12):23702-6.

33. Joergensen MT, Brusgaard K, Novovic S, Andersen AM, Hansen MB, Gerdes AM, et al. Is the SPINK1 variant p.N34S overrepresented in patients with acute pancreatitis? Eur J Gastroenterol Hepatol. 2012;24(3):309-15.

34. Kasap E, Akyildiz M, Tekin F, Akarca U. Angiotensin-converting enzyme genotype and acute pancreatitis in Turkey. Balkan Journal of Medical Genetics. 2009;12(2):39-44.

35. Kim MS, Lee DH, Kang HS, Park HS, Jung S, Lee JW, et al. [Genetic polymorphisms of alcohol-metabolizing enzymes and cytokines in patients with alcohol induced pancreatitis and alcoholic liver cirrhosis]. Korean J Gastroenterol. 2004;43(6):355-63.

36. Kostuch M, Rudzki S, Semczuk A, Kulczycki L. CFTR gene mutations in patients suffering from acute pancreatitis. Med Sci Monit. 2002;8(9):Br369-72.

37. Koziel D, Gluszek S, Kowalik A, Chlopek M. CTRC gene polymorphism (p.G60=; c.180 C > T) in acute pancreatitis. BMC Gastroenterol. 2017;17(1):13.

38. Kume K, Masamune A, Mizutamari H, Kaneko K, Kikuta K, Satoh M, et al. Mutations in the serine protease inhibitor Kazal Type 1 (SPINK1) gene in Japanese patients with pancreatitis. Pancreatology. 2005;5(4-5):354-60.

39. Kume K, Masamune A, Takagi Y, Kikuta K, Watanabe T, Satoh K, et al. A loss-of-function p.G191R variant in the anionic trypsinogen (PRSS2) gene in Japanese patients with pancreatic disorders. Gut. 2009;58(6):820-4.

40. Kuwata K, Hirota M, Sugita H, Kai M, Hayashi N, Nakamura M, et al. Genetic mutations in exons 3 and 4 of the pancreatic secretory trypsin inhibitor in patients with pancreatitis. J Gastroenterol. 2001;36(9):612-8.

41. Li D, Li J, Wang L, Zhang Q. Association between IL-1beta, IL-8, and IL-10 polymorphisms and risk of acute pancreatitis. Genet Mol Res. 2015;14(2):6635-41.

42. Liu YG, Dan G, Wu LJ, Chen GY, Wu AL, Zeng P, et al. Functional Effect of Polymorphisms in the Promoter of TNFAIP3 (A20) in Acute Pancreatitis in the Han Chinese Population. PLoS One. 2014;9(7).

43. Ma M, Zhai CX, Sun CX. Correlations Between LP-PLA2 Gene Polymorphisms and Susceptibility and Severity of Acute Pancreatitis in a Chinese Population. Gastroenterology research and practice. 2017;21(4):206-12.

44. Makhija R, Kingsnorth A, Demaine A. Gene polymorphisms of the macrophage migration inhibitory factor and acute pancreatitis. Jop. 2007;8(3):289-95.

45. Markova EV, Zotova NV, Savchenko AA, Titova NM, Slepov EV, Cherdancev DV, et al. Lymphocyte metabolism in patients with acute pancreatitis and different genotypes of GSTM1 and GSTT1 genes. [Russian]. Biomeditsinskaya Khimiya. 2006;52(3):317-26.

46. Martins FD, Msc BCG, Rodrigues AS, Rueff J. Genetic Susceptibility in Acute Pancreatitis Genotyping of GSTM1, GSTT1, GSTP1, CASP7, CASP8, CASP9, CASP10, LTA, TNFRSF1B, and TP53 Gene Variants. Pancreas. 2017;46(1):71-6.

47. Masamune A, Kume K, Kikuta K, Watanabe T, Hirota M, Satoh K, et al. -651C/T promoter polymorphism in the CD14 gene is associated with severity of acute pancreatitis in Japan. J Gastroenterol. 2010;45(2):225-33.

48. Masamune A, Ariga H, Kume K, Kakuta Y, Satoh K, Satoh A, et al. Genetic background is different between sentinel and recurrent acute pancreatitis. J Gastroenterol Hepatol. 2011;26(6):974-8.

49. Matas-Cobos AM, Redondo-Cerezo E, Alegria-Motte C, Martinez-Chamorro A, Saenz-Lopez P, Jimenez P, et al. The role of Toll-like receptor polymorphisms in acute pancreatitis occurrence and severity. Pancreas. 2015;44(3):429-33.

50. Nijmeijer RM, van Santvoort HC, Zhernakova A, Teller S, Scheiber JA, de Kovel CG, et al. Association Analysis of Genetic Variants in the Myosin IXB Gene in Acute Pancreatitis. PLoS One. 2013;8(12).

51. Nijmeijer RM, Schaap FG, Smits AJ, Kremer AE, Akkermans LM, Kroese AB, et al. Impact of global Fxr deficiency on experimental acute pancreatitis and genetic variation in the FXR locus in human acute pancreatitis. PLoS One. 2014;9(12):e114393.

52. O'Reilly DA, Witt H, Rahman SH, Schulz HU, Sargen K, Kage A, et al. The SPINK1 N34S variant is associated with acute pancreatitis. European Journal of Gastroenterology and Hepatology. 2008;20(8):726-31.

53. Oruc N, Papachristou GI, Avula H, Slivka A, Lamb J, Whitcomb DC. Angiotensin-converting enzyme gene DD genotype neither increases susceptibility to acute pancreatitis nor influences disease severity. Hpb. 2009;11(1):45-9.

54. Ozhan G, Yanar HT, Ertekin C, Alpertunga B. Polymorphisms in tumour necrosis factor alpha (TNFalpha) gene in patients with acute pancreatitis. Mediators Inflamm. 2010;2010:482950.

55. Ozhan G, Yanar TH, Ertekin C, Alpertunga B. The effect of genetic polymorphisms of cyclooxygenase 2 on acute pancreatitis in Turkey. Pancreas. 2010;39(3):371-6.

56. Ozhan G, Sari FM, Vefai M, Yanar HT, Alpertunga B. Acute pancreatitis is associated with Ser608Leu iNOS polymorphism. Folia Biol (Praha). 2012;58(6):256-60.

57. Padureanu V, Boldeanu MV, Streata I, Cucu MG, Silosi I, Boldeanu L, et al. Determination of VEGFR-2 (KDR) 604A>G Polymorphism in Pancreatic Disorders. International journal of molecular sciences. 2017;18(2).

58. Papachristou GI, Sass DA, Avula H, Lamb J, Lokshin A, Barmada MM, et al. Is the monocyte chemotactic protein-1 -2518 G allele a risk factor for severe acute pancreatitis? Clinical gastroenterology and hepatology : the official clinical practice journal of the American Gastroenterological Association. 2005;3(5):475-81.

59. Papachristou GI, Muddana V, Papachristou DJ, Stello K, Whitcomb DC. Epidermal growth factor serum levels and the 61 G/A polymorphism in patients with acute pancreatitis. Dig Dis Sci. 2010;55(9):2676-80.

60. Park JW, Choi JS, Han KJ, Lee SH, Kim EJ, Cho JH. Association of a genetic polymorphism of IL1RN with risk of acute pancreatitis in a Korean ethnic group. Gastroenterology research and practice. 2017.

61. Pezzilli R, Morselli-Labate AM, Mantovani V, Romboli E, Selva P, Migliori M, et al. Mutations of the CFTR gene in pancreatic disease. Pancreas. 2003;27(4):332-6.

62. Polonikov AV, Samgina TA, Nazarenko PM, Bushueva OY, Ivanov VP. Alcohol Consumption and Cigarette Smoking are Important Modifiers of the Association Between Acute Pancreatitis and the PRSS1-PRSS2 Locus in Men. Pancreas. 2017;46(2):230-6.

63. Powell JJ, Fearon KC, Siriwardena AK, Ross JA. Evidence against a role for polymorphisms at tumor necrosis factor, interleukin-1 and interleukin-1 receptor antagonist gene loci in the regulation of disease severity in acute pancreatitis. Surgery. 2001;129(5):633-40.

64. Radosavljevic I, Milojevic A, Miljkovic J, Divjak A, Jelic I, Artinovic V, et al. Lack of PRSS1 and SPINK1 polymorphisms in Serbian acute pancreatitis patients. Serbian Journal of Experimental and Clinical Research. 2015;16(3):201-6.

65. Rahman SH, Salter G, Holmfield JH, Larvin M, McMahon MJ. Soluble CD14 receptor expression and monocyte heterogeneity but not the C-260T CD14 genotype are associated with severe acute pancreatitis. Crit Care Med. 2004;32(12):2457-63.

66. Rahman SH, Ibrahim K, Larvin M, Kingsnorth A, McMahon MJ. Association of antioxidant enzyme gene polymorphisms and glutathione status with severe acute pancreatitis. Gastroenterology. 2004;126(5):1312-22.

67. Rai P, Sharma A, Gupta A, Aggarwal R. Frequency of SPINK1 N34S mutation in acute and recurrent acute pancreatitis. J Hepatobiliary Pancreat Sci. 2014;21(9):663-8.

68. Samgina TA, Bushueva OI, Ivanov VP, Solodilova MA, Nazarenko PM, Polonikov AV. The association study of the promoter polymorphism -308G>A of tumor necrosis factor gene with the development and severity of acute pancreatitis in Russian population of Kursk region. [Russian]. Eksp Klin Gastroenterol. 2014(9):17-20.

69. Samgina TA, Bushueva OY, Nazarenko PM, Polonikov AV. Association of the HindIII Lipoprotein Lipase Gene Polymorphism with the Development of the Non-Biliary Acute Pancreatitis: a Pilot Study. Bulletin of experimental biology and medicine. 2016;161(1):79-82.

70. Sargen K, Demaine AG, Kingsnorth AN. Cytokine gene polymorphisms in acute pancreatitis. Jop. 2000;1(2):24-35.

71. Sharma A, Muddana V, Lamb J, Greer J, Papachristou GI, Whitcomb DC. Low serum adiponectin levels are associated with systemic organ failure in acute pancreatitis. Pancreas. 2009;38(8):907-12.

72. Skipworth JR, Nijmeijer RM, van Santvoort HC, Besselink MG, Schulz HU, Kivimaki M, et al. The effect of renin angiotensin system genetic variants in acute pancreatitis. Annals of surgery. 2015;261(1):180-8.

73. Smithies AM, Sargen K, Demaine AG, Kingsnorth AN. Investigation of the interleukin 1 gene cluster and its association with acute pancreatitis. Pancreas. 2000;20(3):234-40.

74. Snarska J, Cieslinska A, Fiedorowicz E, Jarmolowska B, Sienkiewicz-Szlapka E, Matysiewicz M, et al. Polymorphism in DPPIV Gene in Acute Pancreatitis. International journal of cancer. 2017;46(9):e71-e2.

75. Srivastava P, Shafiq N, Bhasin DK, Rana SS, Pandhi P, Behera A, et al. Differential expression of heat shock protein (HSP) 70-2 gene polymorphism in benign and malignant pancreatic disorders and its relationship with disease severity and complications. Jop. 2012;13(4):414-9.

76. Takagi Y, Masamune A, Kume K, Satoh A, Kikuta K, Watanabe T, et al. Microsatellite polymorphism in intron 2 of human Toll-like receptor 2 gene is associated with susceptibility to acute pancreatitis in Japan. Hum Immunol. 2009;70(3):200-4.

77. Tiszlavicz Z, Szabolcs A, Takacs T, Farkas G, Kovacs-Nagy R, Szantai E, et al. Polymorphisms of beta defensins are associated with the risk of severe acute pancreatitis. Pancreatology. 2010;10(4):483-90.

78. Tukiainen E, Kylanpaa ML, Kemppainen E, Nevanlinna H, Paju A, Repo H, et al. Pancreatic secretory trypsin inhibitor (SPINK1) gene mutations in patients with acute pancreatitis. Pancreas. 2005;30(3):239-42.

79. Tukiainen E, Kylanpaa ML, Puolakkainen P, Kemppainen E, Halonen K, Orpana A, et al. Polymorphisms of the TNF, CD14, and HSPA1B genes in patients with acute alcohol-induced pancreatitis. Pancreas. 2008;37(1):56-61.

80. Tukiainen E, Kylanpaa ML, Repo H, Orpana A, Methuen T, Salaspuro M, et al. Hemostatic gene polymorphisms in severe acute pancreatitis. Pancreas. 2009;38(2):e43-6.

81. Weis S, Jesinghaus M, Kovacs P, Schleinitz D, Schober R, Ruffert C, et al. Genetic analyses of heme oxygenase 1 (HMOX1) in different forms of pancreatitis. PLoS One. 2012;7(5):e37981.

82. Weiss FU, Schurmann C, Guenther A, Ernst F, Teumer A, Mayerle J, et al. Fucosyltransferase 2 (FUT2) non-secretor status and blood group B are associated with elevated serum lipase activity in asymptomatic subjects, and an increased risk for chronic pancreatitis: a genetic association study. Gut. 2015;64(4):646-56.

83. Yang B, O'Reilly DA, Demaine AG, Kingsnorth AN. Study of polymorphisms in the CYP2E1 gene in patients with alcoholic pancreatitis. Alcohol. 2001;23(2):91-7.

84. Yang WG, Wang WP, Zhu CJ, Liu YF, Guo GC, Xue JF. Association of polymorphism variation in interleukin-8 with the risk of developing acute pancreatitis. International Journal of Clinical and Experimental Pathology. 2016;9(11):11958-64.

85. Ye BN, Wang YH, Shi XQ, Wang JL, Long DL, Li K. Association between interleukin-18 genetic polymorphisms and development of acute pancreatitis risk in a Chinese population. International Journal of Clinical and Experimental Pathology. 2016;9(9):9387-93.

86. Zhang D, Li J, Jiang ZW, Yu B, Tang X. Association of two polymorphisms of tumor necrosis factor gene with acute severe pancreatitis. The Journal of surgical research. 2003;112(2):138-43.

87. Zhang DL, Zheng HM, Yu BJ, Jiang ZW, Li JS. Association of polymorphisms of IL and CD14 genes with acute severe pancreatitis and septic shock. World J Gastroenterol. 2005;11(28):4409-13.

88. Zhang D, Zheng H, Zhou Y, Yu B, Li J. TLR and MBL gene polymorphisms in severe acute pancreatitis. Mol Diagn Ther. 2008;12(1):45-50.

89. Zhang C, Guo L, Qin Y, Li G. Interaction between polymorphisms of TLR4 gene G11367C in 3' untranslated region and IkappaB-alpha Hae III in acute pancreatitis and the degree of severity. [Chinese]. Zhong nan da xue xue bao. 2016;Yi xue ban = Journal of Central South University. Medical sciences. 41(3):272-81.

90. Zhang CX, Guo LK, Zhang LL, Qin YM, Chang TM. Interaction of polymorphisms of TNF-alpha gene promoter -308G/A and PPAR-gamma2 gene -C34G with acute pancreatitis and its severity degree. [Chinese]. Journal of Xi'an Jiaotong University (Medical Sciences). 2017;38(1):76-82 and 7.

91. Avanthi SU, Kanth VVR, Agarwal J, Lakhtakia S, Gangineni K, Rao GV, et al. Association of claudin2 and PRSS1-PRSS2 polymorphisms with idiopathic recurrent acute and chronic pancreatitis: A case-control study from India. J Gastroenterol Hepatol. 2015;30(12):1796-801.

92. Bishop MD, Freedman SD, Zielenski J, Ahmed N, Dupuis A, Martin S, et al. The cystic fibrosis transmembrane conductance regulator gene and ion channel function in patients with idiopathic pancreatitis. Hum Genet. 2005;118(3-4):372-81.

93. Kasap E, Akyildiz M, Akarca U. Cholangitis of pancreatitis? Does the angiotensin-converting enzyme genotype favor either? Balkan Journal of Medical Genetics. 2009;12(2):53-7.

94. LaRusch J, Lozano-Leon A, Stello K, Moore A, Muddana V, O'Connell M, et al. The Common Chymotrypsinogen C (CTRC) Variant G60G (C.180T) Increases Risk of Chronic Pancreatitis But Not Recurrent Acute Pancreatitis in a North American Population. Clinical and Translational Gastroenterology. 2015;6.

95. Muddana V, Lamb J, Greer JB, Elinoff B, Hawes RH, Cotton PB, et al. Association between calcium sensing receptor gene polymorphisms and chronic pancreatitis in a US population: role of serine protease inhibitor Kazal 1type and alcohol. World J Gastroenterol. 2008;14(28):4486-91.

96. Muddana V, Park J, Lamb J, Yadav D, Papachristou GI, Hawes RH, et al. Are genetic variants in the platelet-derived growth factor [beta] gene associated with chronic pancreatitis? Pancreas. 2010;39(8):1215-9.

97. Yu W, Ned R, Wulf A, Liu T, Khoury MJ, Gwinn M. The need for genetic variant naming standards in published abstracts of human genetic association studies. BMC research notes. 2009;2:56.

98. Zerbino DR, Achuthan P, Akanni W, Amode MR, Barrell D, Bhai J, et al. Ensembl 2018. Nucleic acids research. 2018;46(D1):D754-D61.

99. Wakefield J. A Bayesian measure of the probability of false discovery in genetic epidemiology studies. Am J Hum Genet. 2007;81(2):208-27.

100. Li X, Song P, Timofeeva M, Meng X, Rudan I, Little J, et al. Systematic meta-analyses and field synopsis of genetic and epigenetic studies in paediatric inflammatory bowel disease. Scientific reports. 2016;6:34076.

101. Patarcic I, Gelemanovic A, Kirin M, Kolcic I, Theodoratou E, Baillie KJ, et al. The role of host genetic factors in respiratory tract infectious diseases: systematic review, meta-analyses and field synopsis. Scientific reports. 2015;5:16119.

102. Montazeri Z, Theodoratou E, Nyiraneza C, Timofeeva M, Chen W, Svinti V, et al. Systematic meta-analyses and field synopsis of genetic association studies in colorectal adenomas. International journal of epidemiology. 2016;45(1):186-205.

103. Ioannidis JP, Boffetta P, Little J, O'Brien TR, Uitterlinden AG, Vineis P, et al. Assessment of cumulative evidence on genetic associations: interim guidelines. International journal of epidemiology. 2008;37(1):120-32.

104. Zhang D, Li J, Jiang Z, Yu B, Tang X, Li W. The relationship between tumor necrosis factor-alpha gene polymorphisms and acute severe pancreatitis. Chin Med J (Engl). 2003;116(11):1779-81.

105. Dianliang Z, Jieshou L, Zhiwei J, Baojun Y. Association of plasma levels of tumor necrosis factor (TNF)-alpha and its soluble receptors, two polymorphisms of the TNF gene, with acute severe pancreatitis and early septic shock due to it. Pancreas. 2003;26(4):339-43.

106. Zhang DL, Li JS, Jiang ZW, Yu BJ, Tang XM, Zheng HM. Association of two polymorphisms of tumor necrosis factor gene with acute biliary pancreatitis. World J Gastroenterol. 2003;9(4):824-8.

107. Zhang D, Li J, Jiang Z, Yu B, Tang X. Significance of tumor necrosis factor-alpha gene polymorphism in patients with acute severe pancreatitis. [Chinese]. Zhonghua yi xue za zhi. 2002;82(22):1529-31.

108. Nie JS, Chen WC. Relationship between genetic polymorphism of MCP-1 and acute pancreatitis in Han population of Suzhou in China. [Chinese]. Chinese Journal of Medical Genetics. 2007;24(5):598-600.

109. Gao HK, Zhang XG, Zhou ZG, Li Y, Chen YD. [Association between toll like receptor 4 (896A>G) mutations and pancreatic necrotic infection in severe acute pancreatitis]. Sichuan da xue xue bao Yi xue ban = Journal of Sichuan University Medical science edition. 2007;38(4):617-9.
